# In Vivo Comparison of the Trueness of Conventional and Digital Interocclusal Registration

**DOI:** 10.3390/bioengineering13101131

**Published:** 2026-09-28

**Authors:** Felix Förtsch, Johannes Ries, Tom Kasperlik, Manfred Wichmann, Ragai Edward Matta

**Affiliations:** Department of Prosthodontics, Erlangen University Hospital, Friedrich-Alexander-University Erlangen-Nuremberg, Glueckstrasse 11, 91054 Erlangen, Germany; johannes.ries@uk-erlangen.de (J.R.); claudia.ehrhardt@uk-erlangen.de (M.W.); ragai.matta@uk-erlangen.de (R.E.M.)

**Keywords:** virtual interocclusal registration, virtual interocclusal record, interocclusal registration, digital impression, intraoral scanner, digital workflow

## Abstract

The objective was to evaluate the trueness of interocclusal registration obtained with three intraoral scanning systems versus a conventional impression and bite registration in fully dentate patients. Twenty adults with complete dentitions and Angle Class I occlusion were enrolled. Complete-arch scans were obtained with Primescan, iTero Element, and TRIOS 4, followed by bilateral virtual interocclusal registration. Conventional impressions and polyvinyl siloxane records were used to fabricate stone casts, digitized with a laboratory scanner with the record and after manual articulation in maximum intercuspation. Industrial scanner datasets served as participant-specific references. Three-dimensional deviations were analyzed by repeated-measures ANOVA and Holm-adjusted paired *t*-tests (α = 0.05). The conventional workflow with an interocclusal record showed the greatest deviation (797 ± 308 μm; *p* < 0.001). Manual articulation (331 ± 167 μm) did not differ significantly from Primescan (262 ± 120 μm), iTero Element (256 ± 101 μm), or TRIOS 4 (325 ± 168 μm) (*p* ≥ 0.223); no differences were found among scanners under the conditions of this study (*p* ≥ 0.468). Virtual interocclusal registration showed greater trueness than the conventional workflow with a physical record, whereas manual articulation performed comparably to digital workflows. These conclusions apply only to fully dentate patients with stable occlusal support and maximum intercuspation.

## 1. Introduction

The increasing digitalization of prosthodontic workflows has substantially changed contemporary restorative dentistry. Intraoral scanning has become an established alternative to conventional impressions, offering advantages in workflow efficiency, patient comfort, data handling, and integration with computer-aided design and manufacturing systems. Recent systematic reviews have shown that digital impressions are at least comparable to conventional techniques for many fixed prosthodontic indications, although their performance remains indication-dependent, particularly in more complex clinical situations [1,2,3].

Beyond the impression itself, the accurate transfer of the maxillomandibular relationship is critical, as inaccuracies in interocclusal registration may lead to articulation errors, occlusal discrepancies, prolonged chairside adjustment, and compromised clinical outcomes [4,5].

Consequently, the reliability of interocclusal records represents a key factor in the successful implementation of fully digital workflows. Current evidence suggests that virtual interocclusal records are promising; however, their accuracy is influenced by multiple technical and clinical variables, including the scanner system, scanning strategy, number and distribution of recorded teeth, extent of the scanned arch, and occlusal conditions. Although some recent clinical studies indicate that digital interocclusal records in dentate patients may be more accurate than conventional methods [6,7], the available evidence is limited and characterized by methodological heterogeneity, making direct comparisons difficult [4,5].

This limitation is particularly relevant because conventional and digital workflows differ fundamentally in their sources of error. Digital workflows depend on scanner-specific data acquisition, alignment algorithms, and clinical recording conditions, whereas conventional methods are prone to material distortion, cast expansion, mounting inaccuracies, and cumulative laboratory errors. The conventional workflow is also influenced by the method used to establish the relationship between the maxillary and mandibular casts. In completely dentate individuals with stable horizontal and vertical occlusal contacts, direct hand articulation of the casts has been reported to reproduce the maximum intercuspation more accurately than registrations obtained by having the participant close through wax or polyvinyl siloxane material [8].

Furthermore, nearly all clinical comparative studies have assessed only the precision of the two techniques [6,7], whereas their trueness has, to date, been evaluated only in an in vitro study [9]. Accordingly, direct comparisons must be performed under controlled clinical conditions.

Therefore, the purpose of this clinical study was to evaluate the trueness of interocclusal registration obtained with 3 intraoral scanning systems, each involving complete-arch intraoral scans of the maxillary and mandibular arches followed by virtual interocclusal registration, and to compare these digital workflows with a conventional impression workflow in which the resulting casts were digitized with a laboratory scanner both with and without a physical interocclusal record. The null hypotheses were that no differences in interocclusal registration trueness would be found (1) between the digital and conventional workflows, (2) between laboratory-scanner digitization of the conventionally produced casts with and without an interocclusal record, and (3) among the 3 intraoral scanning systems.

## 2. Materials and Methods

Twenty healthy adult participants with complete dentitions, Angle Class I occlusion, and no prosthetic restorations were recruited for this study. Participants presenting with gingivitis, periodontitis, temporomandibular disorders, orofacial pain, or known allergies to impression materials were excluded. All participants provided written informed consent prior to enrolment. Participants were fully informed that they could withdraw from the study at any time without providing a reason and without any resulting disadvantage. The study was approved by the Ethics Committee of Friedrich-Alexander-Universität Erlangen-Nürnberg, Germany (Ref. No. 416_18 B), and was registered in the German Clinical Trials Register (DRKS-ID: DRKS00027988). Based on comparable clinical studies, a sample size of 20 participants was considered sufficient to provide meaningful data for the present investigation [6,7,10].

All conventional impressions and intraoral scans were performed by the same clinician. Intraoral scans were obtained using three intraoral scanning systems in no predefined order: Primescan (Dentsply Sirona, Charlotte, NC, USA; CEREC SW 5.0.2), iTero Element (Align Technology, San Jose, CA, USA; software version 1.9), and TRIOS 4 (3Shape, Copenhagen, Denmark; software version 19.2.2). All scanners were calibrated before scanning each participant according to the respective manufacturers’ recommendations. The scanning paths for the maxillary and mandibular arches followed the corresponding manufacturer-recommended protocols. With Primescan, scanning was initiated on the palatal or lingual surface of the most posterior molar and continued along the entire arch to the contralateral side, followed by a return scan along the occlusal surfaces and subsequently along the buccal surfaces. With iTero Element, the palatal or lingual and occlusal surfaces were scanned using a comparable sequence; however, the buccal surfaces were recorded separately from each most posterior molar toward the midline. With TRIOS 4, scanning was initiated on the occlusal surface of a most posterior molar and continued along the entire arch, followed by a return scan along the palatal or lingual surfaces and, subsequently, along the buccal surfaces. During the occlusal scan of the anterior region, a sweeping motion across the incisal edges was performed. Interocclusal registration was performed bilaterally from the first premolar to the second molar. The resulting digitally articulated models were exported as STL files for subsequent analysis.

Conventional impressions were taken using a high-precision polyether impression material (Impregum Penta, 3M Deutschland GmbH, Neuss, Germany). Interocclusal records were obtained using a polyvinyl siloxane bite registration material (Futar D, Kettenbach GmbH & Co. KG, Eschenburg, Germany), which was applied as a continuous strand onto the mandibular dentition. The impressions were poured according to the manufacturer’s instructions using type IV high-strength dental stone (FujiRock EP, GC Europe, Leuven, Belgium). The resulting stone casts were digitized with a laboratory scanner (D1000, 3Shape, Copenhagen, Denmark) both with the corresponding interocclusal record and without an interocclusal record, with the casts manually positioned in maximal intercuspation. All laboratory scanning and manual articulation procedures were performed by the same experienced dental technician, and each procedure was carried out once. The resulting datasets were exported as STL files for further analysis. In addition, the stone casts were digitized in maximum intercuspation using a high-precision industrial scanner (ATOS Triple Scan, Carl Zeiss GOM Metrology GmbH, Braunschweig, Germany) to generate a virtual reference.

To evaluate the trueness of bite registration, the virtual reference model of each participant was first imported into digital inspection software (GOM Inspect Professional 2020, Carl Zeiss GOM Metrology GmbH). Subsequently, the five articulated maxillary and mandibular datasets were imported individually and superimposed onto the reference model to determine deviations from the virtual reference. First, standardized coordinate systems were generated on all teeth of the mandibular reference model using a 3-2-1 alignment based on the cusps and incisal edges of the respective teeth. For all best-fit superimpositions, only the tooth surfaces of the respective arch were used as the alignment area. The mandibular and maxillary teeth were selected once on the reference model, and these predefined reference areas were subsequently used for all comparison datasets. Based on these selections, the best-fit superimposition was calculated automatically by the software, ensuring a standardized and reproducible alignment procedure for each dataset. The dataset to be evaluated was then aligned to the reference model by best-fit superimposition using the mandibular teeth, and the coordinate systems from the reference model were transferred to the mandibular model under evaluation. A second best-fit superimposition was then performed using the maxillary teeth, enabling calculation of the three-dimensional linear deviations between the corresponding mandibular coordinate systems (Figure 1). For statistical analysis, the mean value of all 14 individual deviations was calculated for each dataset. Statistical analyses were performed using R software (version 4.5.2; R Core Team, R Foundation for Statistical Computing, Vienna, Austria), with the level of significance set at α = 0.05. Data normality was assessed using the Shapiro–Wilk test. Differences between groups were analyzed using one-way repeated-measures ANOVA with Greenhouse–Geisser correction, followed by paired *t*-tests with Holm correction for multiple comparisons. A flowchart of the study design is provided in Figure 2.

## 3. Results

Twenty participants (14 women and 6 men) with a mean age of 23.9 ± 2.45 years (range, 21 to 32 years) were included in the analysis. The Greenhouse–Geisser-corrected repeated-measures ANOVA revealed a significant main effect of the registration workflow (F(2.10, 39.50) = 32.40, *p* < 0.001, ηp^2^ = 0.60). The highest mean three-dimensional linear deviation was observed for the conventional method using an interocclusal record for laboratory scanning of the stone casts (797 ± 308 μm). All other study groups demonstrated significantly lower deviations (*p* < 0.001). The conventional workflow involving laboratory scanning of the stone casts positioned manually in maximum intercuspation showed a slightly higher mean deviation (331 ± 167 μm) than the intraoral scanner workflows; however, none of these comparisons reached statistical significance (*p* = 0.223 vs. Primescan; *p* = 0.223 vs. iTero Element; *p* > 0.999 vs. TRIOS 4).

Among the intraoral scanners, iTero Element exhibited the lowest mean deviation (256 ± 101 μm), followed by Primescan (262 ± 120 μm) and TRIOS 4 (325 ± 168 μm). Descriptive statistics for all registration workflows are presented in Table 1. No statistically significant differences were found between the intraoral scanners (iTero Element vs. Primescan: *p* > 0.999; iTero Element vs. TRIOS 4: *p* = 0.468; Primescan vs. TRIOS 4: *p* = 0.468). Figure 3 presents a box-and-whisker plot illustrating the distribution of deviations, including all statistically significant pairwise comparisons. Participant-specific mean deviations are summarized in Appendix A Table A1.

## 4. Discussion

The first null hypothesis was partially rejected. The conventional workflow in which the stone casts were positioned with a polyvinyl siloxane interocclusal record showed significantly greater deviation from the reference than all three digital workflows, whereas the conventional casts positioned manually in maximum intercuspation did not differ significantly from any intraoral scanner workflow. The second null hypothesis was rejected because laboratory-scanner digitization of the casts with an interocclusal record showed significantly lower trueness than digitization after manual positioning in maximum intercuspation. The third null hypothesis was not rejected, as no significant differences were identified among Primescan, iTero Element, and TRIOS 4.

The direction of these findings is consistent with the most directly comparable study that evaluated trueness. In that in vitro investigation, Ries et al. [9] reported a mean deviation of 120 ± 40 μm for completely digital interocclusal registration and 450 ± 470 μm for the conventional workflow with a physical interocclusal record. The corresponding deviations in the present clinical study were higher, ranging from 256 to 325 μm for the intraoral scanner workflows and reaching 797 ± 308 μm for the conventional workflow with an interocclusal record. The difference in magnitude between the present findings and the in vitro results of Ries et al. [9] is plausible because the present protocol incorporated clinical variables that are absent under laboratory conditions, including variability in mandibular closure, patient movement, saliva, and restricted access. The magnitude of the deviations observed in the present study is also consistent with the broader evidence summarized by Morsy and El Kateb [11]. In their systematic review and meta-analysis of static virtual articulation, the pooled mean trueness based on linear deviations was 243.53 μm (95% CI, 144.90 to 342.17 μm). Although the pooled estimate was derived from heterogeneous clinical and in vitro study designs and different methods of assessing trueness and therefore cannot be directly equated with the present three-dimensional coordinate-based analysis, it is within the same range as the deviations observed for the three intraoral scanner workflows in the present study.

The substantially lower deviation obtained when the stone casts were positioned manually rather than with the physical record is clinically relevant. In the present protocol, the polyvinyl siloxane material was applied as a continuous strand before the participant closed. Interposition of a registration material before mandibular closure may interfere with complete intercuspation because of the material’s resistance to closure. Additional errors may occur during subsequent repositioning and seating of the casts in the interocclusal record [8,12,13]. Van den Bergh et al. [8] similarly found significant discrepancies when completely dentate participants closed through wax or polyvinyl siloxane material, whereas a record injected from the buccal aspect after closure did not differ significantly from hand articulation. The present results extend that observation to a three-dimensional assessment of trueness and support direct hand articulation when completely dentate casts have sufficient vertical support and horizontal stability. Similar findings were reported by Zimmermann et al. [14] in an in vivo investigation of 10 individuals with complete dentitions and no dental rehabilitations. No significant differences in translational or rotational parameters were detected between the evaluated digital buccal scan procedures and a cast-based registration method. Although their analytical approach differed from the three-dimensional deviation analysis used in the present study, these findings further indicate that accurate reproduction of maximum intercuspation does not necessarily require an interposed registration material when completely dentate arches provide stable occlusal support. This recommendation should not be extrapolated to dentitions with inadequate occlusal stops, prepared teeth, extensive edentulous spaces, or unstable intercuspation, for which an interocclusal record may remain necessary.

Most previous clinical comparisons have focused on precision or the reproduction of occlusal contacts rather than on trueness. Iwauchi et al. reported smaller discrepancies between repeated digital registrations than between repeated conventional registrations [6], while Morsy and El Kateb found superior precision for digital records in both complete-arch and quadrant conditions [7]. In addition to reproducibility, Abdulateef et al. [10] evaluated the ability of virtual interocclusal records to reproduce occlusal relationships in 10 participants. They reported a sensitivity of 87% and a specificity of 95% for identifying sites of close proximity and clearance, although repeated buccal registrations showed only fair reproducibility. These studies provide clinical evidence regarding the precision and occlusal contact representation of virtual interocclusal records; however, these outcomes are conceptually distinct from trueness. A registration method may be reproducible while still being systematically displaced from the true maxillomandibular relationship. By using a participant-specific reference and quantifying three-dimensional deviations from this reference, the present study therefore extends the available clinical evidence by specifically assessing trueness rather than reproducibility or contact detection alone.

No significant differences were detected among the three intraoral scanning systems under the conditions of this study. Although the scanners use different acquisition technologies and proprietary alignment algorithms, no scanner-dependent effect was detected under the standardized conditions of this study. Bilateral buccal registrations extending from the first premolar to the second molar provided distributed alignment information, and the participants had complete natural dentitions with stable Angle Class I occlusion. The use of a bilateral registration strategy is supported by recent experimental and systematic evidence. Matta et al. [15] compared bilateral buccal, unilateral buccal, and anterior virtual interocclusal registration in a completely dentate in vitro model using 3 intraoral scanners and found significantly greater accuracy with bilateral buccal registration, irrespective of the scanner used. In addition, deviations with unilateral and anterior registrations tended to increase with increasing distance from the registration site. A systematic review by Revilla-León et al. [16] similarly identified the number, location, and extension of occlusal records as relevant factors affecting the accuracy of maximum intercuspation. For complete-arch scans, the authors recommended a bilateral posterior occlusal record incorporating at least 4 teeth, while areas with 2 or more missing teeth were associated with reduced accuracy. The bilateral registrations extending from the first premolar to the second molar used in the present study therefore provided favorable conditions for virtual alignment and may partly explain the relatively consistent results among the three scanner systems. Recent data also indicate that scanner-specific effects may depend on adherence to the respective acquisition protocol. Einhorn et al. [17] evaluated 4 intraoral scanners under manufacturer-recommended and alternative interocclusal scanning protocols. Under the recommended protocols, no significant differences in trueness were identified among TRIOS 3, Primescan, and Smile Scan, whereas the CS3800 showed greater deviation. When alternative protocols were used, scanner-dependent effects became more pronounced; in particular, TRIOS 3 demonstrated a significant increase in trueness error, whereas Primescan remained comparatively stable. These findings suggest that scanner hardware or software should not be considered independently of the acquisition strategy and may help explain why no scanner-dependent effect was detected in the present study, in which the scan paths followed the respective manufacturers’ recommendations and the interocclusal registration procedure was standardized. These favorable conditions may have reduced scanner-dependent effects. The numerical differences among the systems should not be interpreted as evidence of superiority or inferiority because the study was not designed as an equivalence trial and the sample size may have been insufficient to detect smaller differences. In addition, virtual articulation remains sensitive to the scan strategy, extent and distribution of the buccal record, arch length, occlusal morphology, and software algorithms [4,5]. Accordingly, the absence of statistically significant differences among the three scanners in the present study should be interpreted as a finding under the specific standardized conditions investigated rather than as evidence of general equivalence among the tested intraoral scanning systems.

From a clinical perspective, the results do not indicate that every digital workflow is inherently superior to every conventional workflow. Rather, the method used to transfer the maxillomandibular relationship was decisive. The digital workflows avoided the physical bite material and several laboratory transfer steps and demonstrated significantly greater trueness than the conventional workflow using an interocclusal record. However, careful manual positioning of stable, completely dentate casts produced deviations that were not significantly different from those of the intraoral scanner workflows. Thus, either virtual registration or direct hand articulation may be appropriate for patients with stable maximum intercuspation, whereas recording through an interposed polyvinyl siloxane material may increase error under the conditions tested. Importantly, this clinical interpretation is restricted to completely dentate patients with stable occlusal support and should not be extrapolated to partially edentulous patients or to situations with reduced or unstable intercuspation. The available evidence indicates that the presence and distribution of teeth contribute substantially to the accuracy of virtual maxillomandibular alignment [14]. Consequently, the favorable performance of both virtual registration and direct hand articulation observed in the present study is likely related, at least in part, to the stable and completely dentate occlusal conditions of the participants. Whether the observed geometric deviations translate into differences in occlusal adjustment time, restoration quality, or patient-centered outcomes remains to be established.

Several limitations should be considered when interpreting the findings of this study. First, the sample consisted of 20 healthy participants with complete natural dentitions, Angle Class I occlusion, and no prosthetic restorations. The results therefore cannot be generalized to patients with malocclusion, tooth wear, mobility, extensive restorations, partial edentulism, implant-supported prostheses, or reduced occlusal stability. Accordingly, the present findings and the clinical recommendations derived from them should be regarded as applicable only to fully dentate patients with stable occlusal support and reproducible maximum intercuspation. In particular, the comparable performance of direct hand articulation and virtual interocclusal registration observed in this study should not be extrapolated to partially edentulous patients or to situations with compromised or unstable occlusal support. Second, the reference was generated from conventionally produced stone casts positioned manually in maximum intercuspation. Although this approach provided a consistent physical reference, it may contain errors from impression making, stone expansion, cast positioning, and industrial scanning. Because the conventional manual-articulation test group and the reference were derived from the same casts and a similar positioning concept, a degree of shared-source bias favoring this group cannot be excluded. This methodological dependency may have reduced the apparent deviation of this group relative to the reference and should therefore be considered when interpreting the absence of a significant difference between manual articulation and the digital workflows. Third, the measured deviation represented the combined effect of arch acquisition, dataset alignment, and interocclusal registration; these components could not be completely separated. In addition, both the digital and conventional interocclusal registrations were performed only once per participant. Consequently, the repeatability or precision of the individual registration procedures could not be assessed within the present study. Finally, the findings are specific to the scanners, software algorithms, recording extent, impression and registration materials, and laboratory procedures used. No a priori sample size calculation was performed, and the study may therefore have been underpowered to detect smaller differences between the investigated workflows or intraoral scanning systems. Future studies should include larger and more diverse clinical populations, repeated registrations, an independent reference method and direct assessment of occlusal contacts and chairside adjustment requirements.

## 5. Conclusions

Within the limitations of this clinical study, virtual interocclusal registration with each of the 3 intraoral scanning systems, based on complete-arch intraoral scans of the maxillary and mandibular arches followed by virtual interocclusal registration, demonstrated greater trueness than the conventional registration protocol evaluated in this study, which used an interposed polyvinyl siloxane interocclusal record, in fully dentate patients with stable maximum intercuspation. Manual positioning of completely dentate casts in maximum intercuspation did not differ significantly from the digital workflows, and no significant differences were found among the intraoral scanners under the conditions of this study. However, the measured deviations reflected the combined effects of arch acquisition, dataset alignment, and interocclusal registration; therefore, the contribution of interocclusal registration alone could not be isolated. These findings support virtual interocclusal registration and, when stable occlusal support is present, direct hand articulation of stone casts, while suggesting that the specific protocol involving interposition of a polyvinyl siloxane registration material may reduce the trueness of the transferred maxillomandibular relationship under the conditions tested. These findings should not be extrapolated to partially edentulous patients or to clinical situations with reduced or unstable occlusal support.

## Figures and Tables

**Figure 1:**
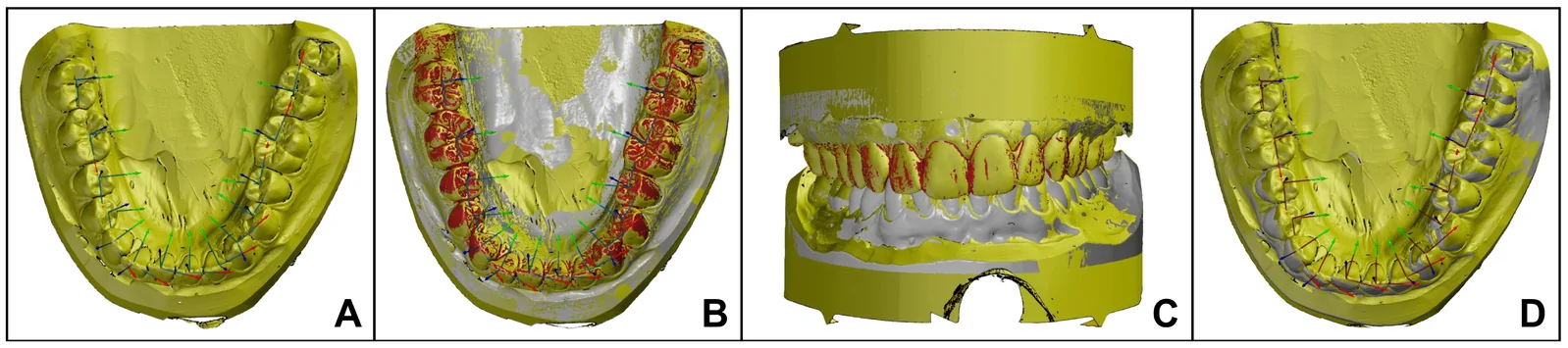
Overview of the matching procedure between the comparison dataset (gray) and the reference dataset (yellow) and subsequent digital analysis. (**A**) Reference dataset with local coordinate systems assigned to each mandibular tooth. (**B**) Best-fit superimposition based on the mandibular teeth. (**C**) Best-fit superimposition based on the maxillary teeth. (**D**) Occlusal view of the final superimposed mandibular datasets with coordinate systems used for determination of positional deviations.

**Figure 2:**
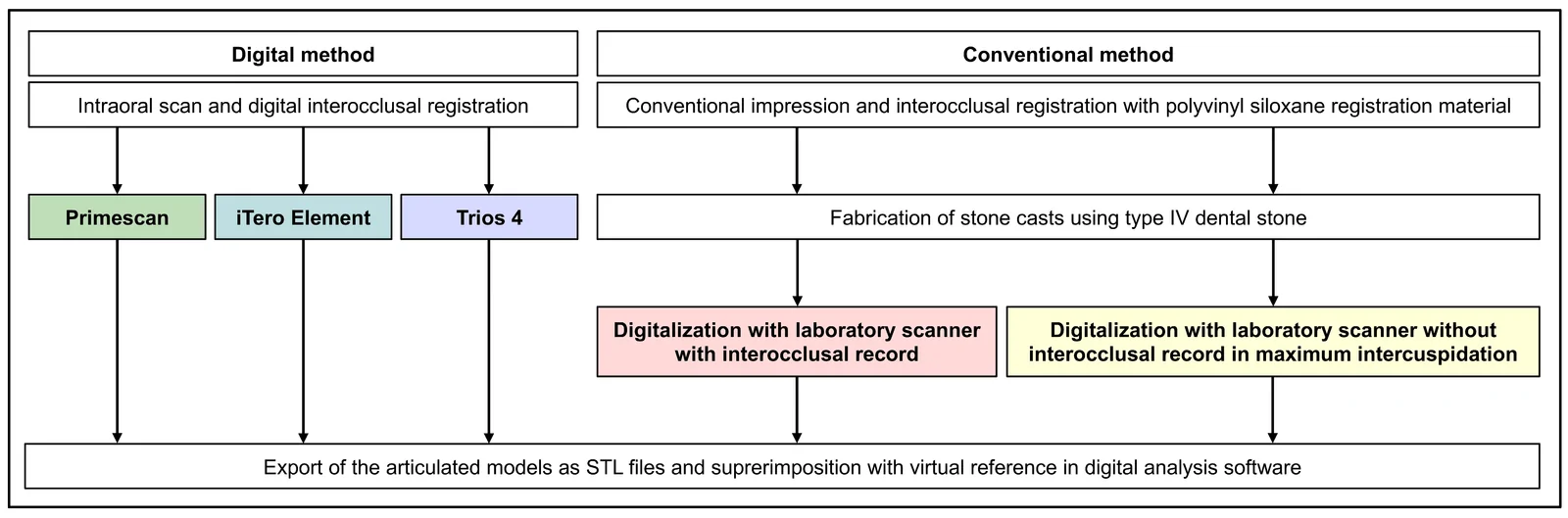
Flowchart of the study design.

**Figure 3:**
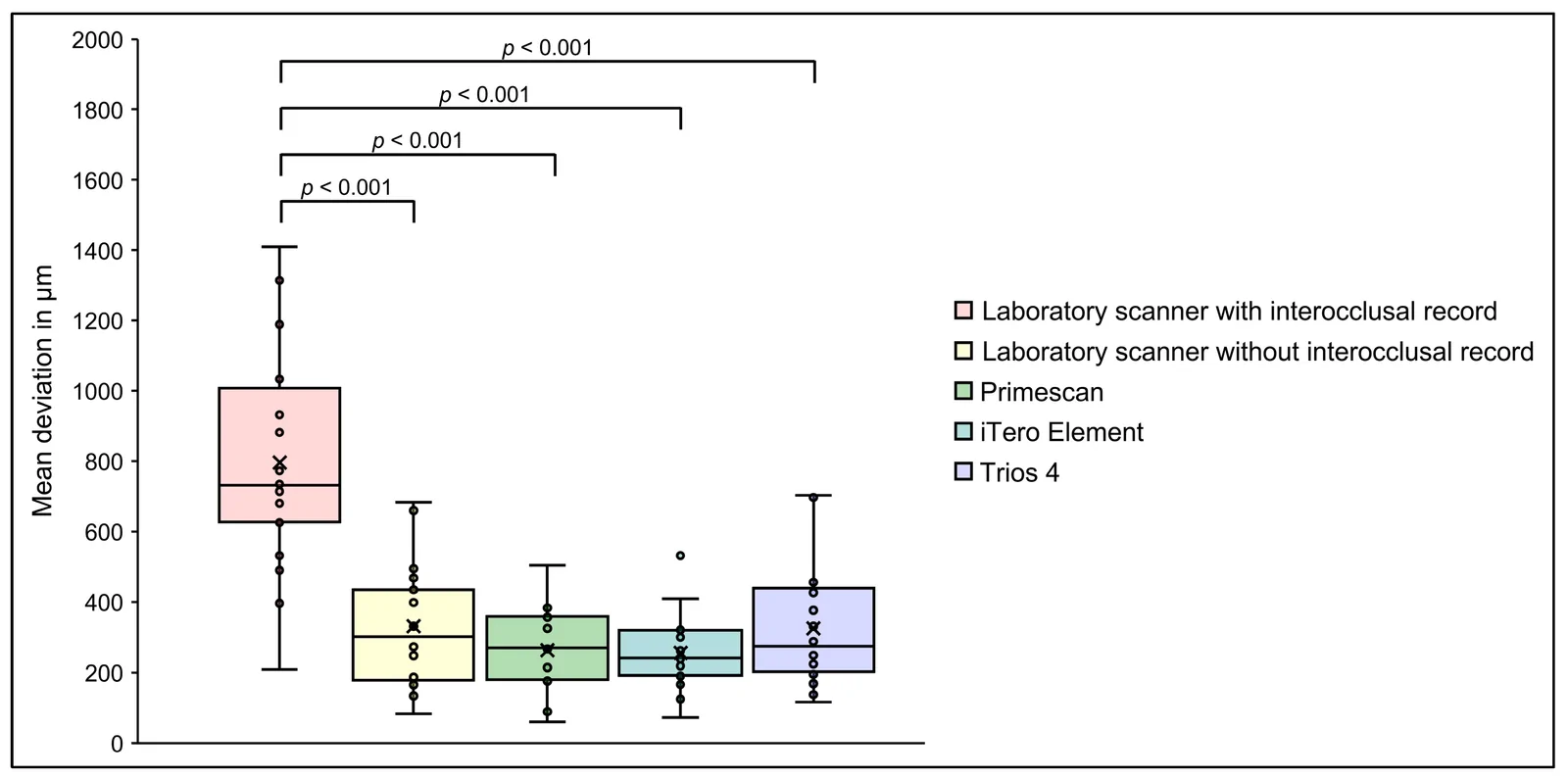
Box-and-whisker plot showing the mean deviation in μm for all registration workflows and the Holm-adjusted *p* values for all statistically significant pairwise comparisons.

**Table 1:** Descriptive statistics for all registration workflows. Mean deviation, standard deviation (SD), 95% confidence interval (95% CI), and minimum (Min) and maximum (Max) deviations, all expressed in μm.

Registration Workflow	Mean [μm]	SD [μm]	95% CI [μm]	Min [μm]	Max [μm]
Lab scanner with interocclusal record	797	308	653–941	209	1410
Lab scanner without interocclusal record	331	167	253–409	83	684
Primescan	262	120	206–318	60	504
iTero Element	256	101	208–303	73	532
Trios 4	325	168	246–404	116	703

## Data Availability

The original contributions presented in this study are included in the article. Further inquiries can be directed to the corresponding author.

## Appendix A

**Table A1:** Mean deviation in μm for all registration workflows for all study participants.

Participant	Mean Deviation in μm				
	Laboratory Scanner with Interocclusal Record	Laboratory Scanner Without Interocclusal Record	Primescan	iTero Element	Trios 4
1	735	341	267	181	233
2	734	264	96	225	288
3	715	435	267	320	242
4	1410	176	214	230	262
5	625	247	326	239	249
6	1195	684	359	242	141
7	731	331	217	242	137
8	532	134	325	199	462
9	396	660	396	320	331
10	1314	272	60	125	224
11	681	257	177	190	697
12	773	83	190	219	116
13	634	398	504	532	456
14	490	166	89	261	196
15	209	177	97	73	376
16	729	495	274	410	169
17	932	409	372	167	426
18	1034	187	272	321	703
19	1189	468	357	300	347
20	882	435	383	318	444
